# Surgery versus conservative treatment in patients with type A distal radius fractures, a randomized controlled trial

**DOI:** 10.1186/1471-2474-15-90

**Published:** 2014-03-19

**Authors:** Monique MJ Walenkamp, J Carel Goslings, Annechien Beumer, Robert Haverlag, Peter A Leenhouts, Egbert JMM Verleisdonk, Ronald SL Liem, Jan Bernard Sintenie, Maarten WGA Bronkhorst, Jasper Winkelhagen, Niels WL Schep

**Affiliations:** 1 Trauma Unit, Department of Surgery, Academic Medical Centre, University of Amsterdam, P.O. Box 22660, Amsterdam, DD 1100, The Netherlands; 2 Department of Orthopedics, Amphia Hospital, P.O. Box 90158, Breda, RK 4800, The Netherlands; 3 Department of Surgery, Onze Lieve Vrouwe Hospital, P.O. Box 95500, Amsterdam, HM 1090, The Netherlands; 4 Department of Surgery, Zaans Medical Centre, P.O. Box 210, Zaandam, EE 1500, The Netherlands; 5 Department of Surgery, Diakonessenhuis, P.O. Box 80250, Utrecht, TG 3508, The Netherlands; 6 Department of Surgery, Groene Hart Hospital, Bleulandweg 10, Gouda, HH 2803, The Netherlands; 7 Department of Surgery, Elkerliek Hospital, P.O. Box 98, Helmond, AB 5700, The Netherlands; 8 Department of Surgery, Bronovo Hospital/MC Haaglanden, P.O. Box 96900, The Hague, JH 2509, The Netherlands; 9 Department of Surgery, Westfriesgasthuis, P.O. Box 600, Hoorn, AR 1620, The Netherlands

**Keywords:** Distal radius fractures, Displaced, Dislocated, Open reduction internal fixation, Volar locking plate, Closed reduction, Plaster immobilization, Randomized controlled trial, Trauma surgery, Wrist injury, Wrist function, Disability arm shoulder hand score, Patient reported outcome, Prehensile grip strength

## Abstract

**Background:**

Fractures of the distal radius are common and account for an estimated 17% of all fractures diagnosed. Two-thirds of these fractures are displaced and require reduction. Although distal radius fractures, especially extra-articular fractures, are considered to be relatively harmless, inadequate treatment may result in impaired function of the wrist. Initial treatment according to Dutch guidelines consists of closed reduction and plaster immobilisation. If fracture redisplacement occurs, surgical treatment is recommended. Recently, the use of volar locking plates has become more popular. The aim of this study is to compare the functional outcome following surgical reduction and fixation with a volar locking plate with the functional outcome following closed reduction and plaster immobilisation in patients with displaced extra-articular distal radius fractures.

**Design:**

This single blinded randomised controlled trial will randomise between open reduction and internal fixation with a volar locking plate (intervention group) and closed reduction followed by plaster immobilisation (control group). The study population will consist of all consecutive adult patients who are diagnosed with a displaced extra-articular distal radius fracture, which has been adequately reduced at the Emergency Department. The primary outcome (functional outcome) will be assessed by means of the Disability Arm Shoulder Hand Score (DASH). Secondary outcomes comprise the Patient-Rated Wrist Evaluation score (PRWE), quality of life, pain, range of motion, radiological parameters, complications and cross-overs. Since the treatment allocated involves a surgical procedure, randomisation status will not be blinded. However, the researcher assessing the outcome at one year will be unaware of the treatment allocation. In total, 90 patients will be included and this trial will require an estimated time of two years to complete and will be conducted in the Academic Medical Centre Amsterdam and its partners of the regional trauma care network.

**Dicussion:**

Ideally, patients would be randomised before any kind of treatment has been commenced. However, we deem it not patient-friendly to approach possible participants before adequate reduction has been obtained.

**Trial registration:**

This study is registered at the Netherlands Trial Register (NTR3113) and was granted permission by the Medical Ethical Review Committee of the Academic Medical Centre on 01-10-2012.

## Background

Fractures of the distal radius account for an estimated 17% of all fractures diagnosed [1,2]. Two-thirds of these fractures are displaced and require reduction [3]. Although extra-articular distal radius fractures are considered to be relatively harmless, inadequate treatment may result in severely impaired function of the wrist [4,5]. The consequences of post-traumatic loss of function are comprehensive, both on individual and societal level, and have long been underestimated [6].

Several treatment modalities to obtain and maintain reduction exist and decision-making is mainly based on fracture type, region and surgeon’s preference [7]. Although good results have been described for both conservative and surgical management, the ideal treatment method remains unknown.

According to current Dutch guidelines, standard treatment for patients with displaced extra-articular distal radius fractures consists of closed reduction and cast immobilisation for four to six weeks [8]. Nevertheless, redisplacement occurs in up to 60% of cases and functional recovery is frequently poor [9-11]. If fracture redisplacement occurs, surgical reduction and fixation is the treatment of choice [8].

A well-established and widely applied surgical approach is open reduction and internal fixation (ORIF). This procedure involves surgical (open) fracture reduction and internal fixation by means of locking plates. Over the past years, the use of volar locking plates has become increasingly popular [12]. This type of osteosynthesis requires a relatively simple volar approach to the wrist, followed by fracture fixation using fixed angle implants [13]. The technique allows more accurate reduction and immediate stable fixation. Subsequent removal of the plate is rarely necessary [7,14]. The fracture stability allows for early mobilisation and may therefore result in an improved recovery of function [7,14]. By 1987 already, Dias et al. concluded that patients who were encouraged to mobilise their injured wrist from the start in a modified cast which only restricted extension, recovered function more quickly than those whose who were immobilised in a conventional plaster cast [10].

A recent randomised controlled trial by Arora et al. compared open reduction and internal fixation with a volar locking plate with closed reduction and plaster immobilisation. They included patients of 65 years and older who had suffered all types of displaced distal radius fractures with inadequate reduction or redisplacement [15]. The operative treatment group showed better wrist function in the early post-operative period. However, at six and twelve months there were no significant differences in wrist function between treatment groups. At all times, grip strength was significantly better in the operative group. These results are consistent with a previous retrospective cohort study among elderly patients conducted by Arora et al. as well [16]. Future studies compare the quality of life between patients treated with a volar locking plate or closed reduction and plaster immobilisation [17].

Despite the high incidence of displaced distal radius fractures and the substantial possible implications of suboptimal management, no high level evidence regarding the best treatment method yet exists. To our knowledge, no studies have been performed comparing conservative treatment with ORIF in patients of all ages with displaced extra-articular distal radius fractures. Therefore, we are proposing to conduct a randomised controlled trial to compare the functional outcome, assessed with the Disability Arm Shoulder Hand Score (DASH), after ORIF with a volar locking plate with closed reduction followed by plaster immobilisation, in patients with displaced extra-articular distal radius fractures. We hypothesise that surgical reduction will result in a more rapid recovery and better functional results at one year follow up than conservative treatment consisting of closed reduction and plaster immobilisation.

The aim of this study is to compare two treatment methods for patients with displaced extra-articular distal radius fractures regarding functional outcome at one year follow up. These treatment methods include open reduction and internal fixation (ORIF) with a volar locking plate and closed reduction followed by plaster immobilisation.

## Methods/Design

This single blinded randomised controlled trial will randomise between open reduction and internal fixation (ORIF) with a volar locking plate (intervention group) and closed reduction followed by plaster immobilisation (control group).

### Participants

The eligible study population will consist of all consecutive adult patients who are diagnosed with a displaced extra-articular distal radius fracture, which has been adequately reduced at the Emergency Department of the Academic Medical Centre Amsterdam or one of the other participating hospitals.

### Inclusion criteria

• Patients ≥ 18 years and ≤ 75 years

• Extra-articular (AO type A) displaced distal radius fracture, as classified on lateral, posterior-anterior and lateral carporadial radiographs by a radiologist or trauma surgeon.

• Acceptable closed reduction obtained according to current Dutch guidelines [1].

•<15° dorsal or <20° volar angulation of the distal fracture fragment

•<5 mm loss of radial height

•≥15° radial inclination

### Exclusion criteria

• Open distal radius fractures

• Multiple trauma patients (Injury Severity Score (ISS) ≥16)

• Other fractures of the affected extremity

• Patients who indicate to have had impaired wrist function prior to injury, for example due to rheumatoid arthritis, neurological disorders of the upper limb or previous malunions in the affected limb.

• Patients suffering from disorders of bone metabolism known to adversely effect fracture healing, such as osteomalacia.

• Patients suffering from connective tissue or (joint) hyperflexibility disorders known to adversely effect fracture healing and/or soft tissue and wound healing.

• Patients unable to understand the treatment information and informed consent forms as judged by the attending physician.

### Interventions

All patients will initially be treated with closed reduction and cast immobilisation. This will take place under local anaesthesia by means of a haematoma block with 20 cc Lidocaine 1%. Closed reduction will be performed according to the Robert-Jones method [18]. This involves increasing the deformity first, then applying continuous traction and immobilising wrist and hand in the reduced position. Additional radiographs will be performed to verify the quality of the reduction (see Inclusion criteria). After this has been confirmed, the wrist will be immobilised according to Dutch guidelines: a dorsal splint for one week. Once informed consent is obtained, patients will be randomized at one week between open reduction and internal fixation with a volar locking plate, or continuation of cast immobilization.

The intervention group will be treated with open reduction and internal fixation with a volar locking plate. The surgery will be performed by a general, trauma or orthopaedic surgeon. In order not to disturb clinical practise, and to provide an accurate comparison of two treatment modalities as they are applied in clinical practise, no specific interval to surgery is prescribed. According to the current standard treatment protocol, antibiotic prophylaxis will be administered pre-operatively. The distal radius will be approached according to Henry, which involves an incision between the tendon of the flexor carpi radialis and the radial artery. The advantage of this approach is the possibility of an easy extension to the proximal or distal part of the forearm and the fact that the plate will be optimally covered by soft tissue [19]. Moreover, the median nerve is not at risk using this technique. After the fracture site is exposed, the fracture will be reduced and provisionally fixed with K-Wires and/or reduction forceps. An appropriate volar locking plate which best suits the anatomy of the wrist and the fracture type will be selected. Screw placement and fracture reduction will be confirmed intra operatively by radiographic images. Wound closure will be performed at the discretion of the surgeon using standard techniques and no post-operative fixation or immobilisation will be applied. During the first follow up visit at five to ten days, wound inspection will be performed. Patients will be instructed to use the affected extremity in daily activities as pain allows.

The control group will continue treatment with cast immobilisation according to Dutch guidelines: a circular cast for another four weeks [8]. At one week and three weeks following initial immobilisation, radiographs will be performed in both groups to ensure that loss of reduction has not occurred. Loss of reduction is defined as: >15° dorsal or >20° volar angulation of the distal fracture fragment, >5 mm loss of radial height, or ≤15° radial inclination [8]. If this is the case, operative treatment will be offered. According to Dutch treatment standards, vitamin C 500 milligrams will be prescribed to all patients at initial presentation and for a duration of two months in order to prevent Complex Regional Pain Syndrome [8].

### Randomisation

All patients diagnosed with an extra-articular AO type A1, A2 or A3 distal radius fractures will be requested to participate in this study. Patients will be eligible after adequate reduction of the fracture has been acquired. Upon obtaining informed consent, patients will be randomised into either the intervention group (ORIF with a volar locking plate) or the control group (closed reduction and plaster immobilisation). This will be performed online by randomisation software provided by the Clinical Research Unit of the Academic Medical Centre Amsterdam. In order to avoid any imbalances between treatment groups, patients will be randomised into three strata according to age: 18-30, 31-60 and >60 years using a block randomisation.

### Blinding

Randomisation status will not be blinded since the treatment allocated involves a surgical procedure.

### Primary outcome

The primary endpoint of this study is wrist function, pain and disability as measured with the DASH score at one year follow up [20]. The Disabilities of the Arm, Shoulder and Hand (DASH) score is a 30-item, self-report questionnaire designed to measure physical function and symptoms in patients with any or several musculoskeletal disorders of the upper limb. The DASH outcome measure is scored in two components: the Disability/Symptom and the optional high performance Sport/Music module. The DASH Disability/Symptom score is a summation of the responses to 11 questions on a scale of 1 to 5, with 0 (no disability) to 100 (severe disability). The questions test the degree of difficulty in performing a variety of physical activities because of arm, shoulder, or hand problems (6 items). It also investigates the severity of pain, tingling (2 items), as well as the effect of the upper limb problem on social activities, work, and sleep (3 items).

### Secondary outcomes

• Rist pain and disability expressed as change on Patients-Rated Wrist Evaluation Score (PRWE). The PRWE is a validated tool for assessing functional outcome in patients with distal radius fractures [21]. This score was first described in 1998 by McDermid et al. [22] and developed by expert surveys. The PRWE is a 15-item questionnaire designed to measure wrist pain and disability in activities of daily living. The PRWE allows patients to rate their levels of wrist pain and disability from 0 to 10, and consists of three subscales: Pain, Function and Cosmetics.

• Quality of Life assessed using the Short Form–36 (SF-36^®^) questionnaire. The SF-36 is a validated multipurpose, short form health survey which contains 36 questions representing eight different health domains [23]. These domains are combined into a mental and physical component scale. From each domain, scores ranging from 0 to 100 points are derived, with lower scores indicating poorer quality of life.

• Pain as indicated on a visual Analogue Scale (VAS), in which 0 implies no pain and 10 the worst possible pain. Patients will be asked to give an estimation of the type and quantity of pain medication taken during all follow up visits.

• Patient satisfaction at one year by simply asking patients if they are satisfied with the result (yes/no).

• Range of motion of the wrist measured on both sides with a handheld goniometer.

• Prehensile grip strength as measured with a Baseline dynamometer.

• Radiological parameters: radial inclination, volar/dorsal tilt, communition, ulnar variance and radial length measured digitally in the Picture Archiving and Communication System (PACS) on standard posterior anterior (PA), lateral carporadial and lateral X-rays of the wrist. Radiographs will be obtained according to standardised procedures. PA radiographs with the shoulder in 90 degrees abduction, elbow in 90 degrees flexion and the wrist in neutral position; lateral X-rays with the shoulder in neutral position and elbow in 90 degrees flexion; and the lateral carporadial radiographs will be obtained by positioning the lower arm on a 20-25 degrees angled wedge.

• Rate of cross-overs

• Complications such as: loss of reduction, fracture malunion or non-union, wound and/or plate infection, tendon irritation and/or rupture, neuropathy and the occurrence of complex regional pain syndrome according to the criteria by Veldman et al. [24].

### Side-effects reporting

All adverse events will be described in patient file during consult at any of the follow-up visits or any other moment if indicated or requested by the patient. Serious adverse events will be reported through the web portal *ToetsingOnline* to the Medical Ethical Review Committee of the Academic Medical Centre of the University of Amsterdam, which approved the protocol, within 15 days after the sponsor has first knowledge of the serious adverse reactions.

### Data collection and follow up

Baseline characteristics will be obtained after randomisation but before treatment takes place. During follow up patients will be asked to return to the hospital for follow up at; one, three and six weeks and three, six and twelve months, according to standard Dutch protocols [8]. During these visits patients will be asked about any complaints and/or complications and physical and radiological examination will be performed. For details, see Table 1. Procedures additional to standard care are highlighted.

**Table 1:** Follow-up visits

**Follow-up at:**	**Tests:**
1 week	VAS, X-ray
3 weeks	VAS, X ray
6 weeks	VAS, ROM, Grip strength, QoL, X-ray, PRWE, DASH
3 months	VAS, ROM, Grip strength, QoL, X-ray, PRWE, DASH
6 months	VAS, ROM, Grip strength, QoL, X-ray, PRWE, DASH
12 months	VAS, ROM, Grip strength, QoL, X-ray, PRWE, DASH

Examinations additional to stand care are bold.

### Sample size

This sample size calculation is based on the primary endpoint, the DASH score. The DASH score of an individual without any complaints of the wrist is 0. The mean DASH score after closed reduction and cast treatment after one year of follow up is 19 with a standard deviation (SD) of 18 [25]. This figure was measured in a patient population in which 72% suffered from a displaced extra-articular distal radius fracture. We assume that treatment with volar plating will decrease the DASH score which is achieved by conservative cast treatment by 15 points, from 19 to 4. Therefore at α = 0.05% and a power of 90%, with an estimated lost to follow-up of 10%, we would require 66 patients in total and 33 per treatment arm, to participate in the trial. This figure was calculated using the standard formula for means of superiority trials: n = [A + B]^2^ * 2 * SD^2^/DIFF^2^, where N = the number of patients required per arm, A the level of significance, B the power, SD the standard deviation of the primary outcome and DIFF the difference between the means. For safety measures and to correct for natural deaths, 45 patients in each arm will be included. From a separate study being conducted at the Academic Medical Centre Amsterdam and two other teaching hospitals, it was established that of the 703 distal radius fractures encountered in one year, 328 were an AO type A2 and A3. Therefore we estimate that we require a maximum of two years to include and follow up the patients in this trial.

### Statistical analysis

Patients will be analysed according to the intention-to-treat protocol. General descriptive statistics on patient characteristic at baseline will be performed including factors such as gender and age. The primary outcome, DASH at one year, will be corrected for age and assessed using an analysis of co-variance (ANCOVA). Trends in DASH scores among the different time points will be assessed using a repeated measures ANOVA. The secondary outcomes; PRWE, quality of life (QoL SF-36), pain as indicated on a Visual Analogue Scale (VAS) and Range of Motion (ROM) will be analysed in a similar manner. The radiological outcome, number of conversions and complication rate will be determined using either a Fisher Exact of a Chi square test, depending on the order of magnitude of the results. Subgroup analyses with regard to DASH score will be performed for gender and age for each randomisation stratum.

### Ethical considerations

This study was approved by the Medical Ethical Review Committee of the Academic Medical Centre of the University of Amsterdam (reference number: project 2012_228).

### Regulation statement

This study will be conducted according to the principles of the Declaration of Helsinki version 59, October 2008 and in accordance with the Medical Research Involving Human Subjects Act (WMO) and other guidelines, regulations and Acts.

### Recruitment and informed consent

Patients diagnosed with a displaced extra-articular distal radius fracture will be approached by the investigators and informed about this trial. Patients will receive an elaborate information sheet and contact details of both the investigator and an independent physician. Possible participants will have a period of reflection of five working days. If a patient decides to participate, written and oral informed consent will be obtained.

### Benefits and risks assessment, group relatedness

The treatment that patients will receive is a component of the standard treatment of care, which currently depends on the surgeon’s preference and the complexity of the fracture. Patients will be asked to return to the hospital for follow up at one, three and six weeks, three months, six months and at twelve months. All visits are part of standard care following a fracture treated in this hospital. During these visits patients will be asked about any complaints and/or complications and physical examination will be performed. The assessment of the range of motion of the wrist will take approximately five minutes. Additional to standard care, patients will be asked to fill out three questionnaires at six weeks, three months, six months and one year. Patients will be asked to fill out a DASH form, rate their pain on a Visual Analogue Scale and give an estimation of the type and quantity of pain medication taken during all visits. This will take approximately ten minutes of their time. The PRWE score and the SF-36 will approximately take another ten minutes each. Subjects could experience mild discomfort during physical examination and testing, but this will be no different from that experienced during physical examination during routine follow-up. X-rays will be taken during every visit of which only the final radiographs at one year are additional to standard care. The burden experienced regarding time spent is difficult to estimate but will most likely not exceed 30 minutes. In the total duration of this study, patients will spend an approximate 150 minutes more. The risks are comparable to those that the standard treatment involves. This comprises the standard risk for undergoing a surgical procedure, including risks related to anaesthesia, neurovascular damage and post-operative wound infection. The risks of plaster immobilisation include redisplacement, malunion, loss of function, carpal tunnel syndrome and complex regional pain syndrome. Close follow up and a protocol of treatment, identical to the standard one, will be applied in every subject. Reduction of risks will be done according to inclusion and exclusion criteria. If complications arise, the treating physician will proportionate the adequate treatment according to the current protocols of treatment based on the published literature.

Subjects can leave the study at any time for any reason if they wish to do so without any consequences. The investigator can decide to withdraw a subject from the study for urgent medical reasons. This study will be terminated prematurely if and when patients experience an amount of discomfort or adverse events that is disproportionate to the benefit of the study and presents too great a risk to the participating study subjects. Since the allocated treatment is part of standard treatment of care, no interim analysis, stopping rules or data monitoring was constructed.

### Indemnities

The institutional review board at the AMC has waived liability insurance, because no additional risk can be attributed to participation in this study.

### Publication plan

The principal investigator, the study designer and the study coordinator will be named author. There will be a limit of ten authors. All others will obtain group authorship in the study group. All authors including group members are allowed to present the results.

### Funder

Academic Medical Centre Graduate School with a PhD Scholarship.

Start date: 26-10-2012.

Intended date of completion: 01-11-2014.

Reporting date: 01-11-2015.

## Discussion

The exact moment of inclusion and randomisation of patients with displaced distal radius fractures has proven to be a complicated issue during the design of this trial. Ideally, patients would be randomised before any kind of treatment has been commenced. However, we deem it unethical and moreover not patient-friendly to approach possible participants before adequate reduction has been obtained. Therefore, after careful collaboration and discussion, the research group has decided upon its current format.

## Abbreviations

AMC: Academic medical centre Amsterdam; ANCOVA: Analysis of covariance; ANOVA: Analysis of variance; AO: Arbeitsgemeinschaft für Osteosynthesefragen; CT: Computed tomography; DASH: Disability arm shoulder hand; ED: Emergency department; GRC: Global rating of change; ISS: Injury severity score; ORIF: Open reduction and internal fixation; PA: Posterior anterior; PACS: Picture archiving and communication system; PRWE: Patient rated wrist evaluation; RH: Radial height of radial length; ROM: Range of motion; QoL: Quality of life; SD: Standard deviation; SF-36: Short form 36; VAS: Visual analogue scale.

## Competing interests

The authors declare that they have no competing interests. No external funding was received for this study.

## Authors’ contributions

All authors participated in the design of the study and the drafting of the manuscript. MMJW: Participated in the design of the study and drafted the manuscript. JCG: Participated in the design of the study and helped to draft the manuscript. AB: Helped to draft and critically revise the manuscript. Made and will make a substantial contribution to acquisition of data. RH: Helped to draft and critically revise the manuscript. Made and will make a substantial contribution to acquisition of data. PAL: Helped to draft and critically revise the manuscript. Made and will make a substantial contribution to acquisition of data. EJMMV: Helped to draft and critically revise the manuscript. Made and will make a substantial contribution to acquisition of data. RSLL: Helped to draft and critically revise the manuscript. Made and will make a substantial contribution to acquisition of data. JBS: Helped to draft and critically revise the manuscript. Made and will make a substantial contribution to acquisition of data. MWGAB: Helped to draft and critically revise the manuscript. Made and will make a substantial contribution to acquisition of data. JW: Helped to draft and critically revise the manuscript. Made and will make a substantial contribution to acquisition of data. NWLS: participated in the design of the study and drafted the manuscript. All authors have read and approved the final manuscript.

## Pre-publication history

The pre-publication history for this paper can be accessed here:

http://www.biomedcentral.com/1471-2474/15/90/prepub
